# Augmentation of Deficient Bone Healing by Pulsed Electromagnetic Fields—From Mechanisms to Clinical Outcomes

**DOI:** 10.3390/bioengineering11121223

**Published:** 2024-12-03

**Authors:** Amr Kaadan, Simona Salati, Stefania Setti, Roy Aaron

**Affiliations:** 1 Department of Orthopedic Surgery, Warren Alpert Medical School of Brown University, Providence, RI 02903, USA; amro_kaadan@alumni.brown.edu; 2 Medical Division, Igea S.p.A, 41012 Carpi, Italy; s.salati@igeamedical.com (S.S.); s.setti@igeamedical.com (S.S.)

**Keywords:** pulsed electromagnetic field, bone repair, intracellular second messenger, adenosine receptor

## Abstract

Pulsed Electromagnetic Fields (PEMF) are widely used, with excellent clinical outcomes. However, their mechanism of action has not yet been completely understood. The purpose of this review is to describe current observations on the mechanisms of PEMF, together with its clinical efficacy. Osteoblast responsiveness to PEMF is described on several scales, from the cell membrane to clinically relevant bone formation. PEMF has been shown to activate membrane adenosine receptors. The role of adenosine receptors in activating intracellular second messenger pathways, such as the canonical Wnt/β-catenin pathway and the mitogen-activated protein kinases (MAPK) pathway, is described. The responsiveness of osteoblasts and the synthesis of structural and signaling proteins constitute the role of PEMFs in promoting osteogenesis and bone matrix synthesis, and they are described. Multiple studies, ranging from observational and randomized to meta-analyses that investigate the clinical efficacy of PEMF, are described. This review presents a favorable conclusion on the clinical effects of PEMF while unlocking the “black box” of PEMF’s mechanism of action, thus improving confidence in the clinical utility of PEMF in bone repair.

## 1. Introduction

Pulsed electromagnetic fields (PEMF) are regarded as safe and efficacious treatments for fracture non-unions and bone defects. Despite excellent clinical outcomes, however, the mechanisms of PEMF stimulation of bone have, until recently, been incompletely understood, compromising confidence in clinical and physiological observations. Within recent years, the mechanisms of PEMF effects on osteoblasts and repairing bone have been elucidated, allowing an understanding of the biology of PEMF-augmented bone formation and repair [1,2]. This review describes cell recognition of PEMF through signal transduction of adenosine A_2A_ and A_3_ cell membrane receptors [3,4] and traces intracellular signaling through pathways such as the WNT–β-catenin pathway [5]. PEMF effects on osteoblast responses of the synthesis of structural and signaling cytokines and the formation of bone matrix are described. Then, a compilation of the clinical results of PEMF treatment of fractures and non-unions through individual studies and meta-analyses is presented [6,7,8]. Understanding of PEMF membrane reception and of the intracellular pathways involved, culminating in the synthesis of extracellular matrix proteins and bone restoration, should enhance confidence in the clinical use of PEMF and the identification of clinical conditions likely to be favorably affected by PEMF exposure. This review describes mechanistic observations of PEMF on bone on several scales, from the cellular to the organismal and clinical levels.

## 2. Cell Reception of Pulsed Electromagnetic Fields—The Role of A_2A_ Adenosine Receptors in PEMF-Mediated Bone Healing

PEMFs have been known to stimulate bone healing for more than 40 years. The most commonly accepted hypothesis of the mechanism of action is that PEMFs act through the modulation of Ca^2+^ intracellular concentration and the action on membrane receptors. Early studies showed that PEMF exposure affects membrane-associated second messenger systems such as Ca^2+^, cAMP, or phosphatidylinositol metabolism and subsequent cellular responses. Cain et al. demonstrated that PEMFs inhibit the cAMP response to parathyroid hormone in bone cells [9]. Similarly, Cadossi et al. found that PEMF exposure enhances the response of human lymphocytes to phytohemagglutinin (PHA) by enhancing ligand-receptor migration and capping at the cell membrane [10]. In 1999, Ventura et al. showed that PEMF-induced nuclear PKC activation led to myocardial opioid gene expression [11].

Adenosine is an endogenous purine nucleoside involved in various physiopathological processes. Adenosine is primarily synthesized through the dephosphorylation of ATP, ADP, and AMP by two hydrolyzing enzymes: ectonucleoside triphosphate diphosphohydrolase (CD39) and ecto-5′-nucleotidase (CD73) [12]. Adenosine functions are mediated by its interactions with four G-protein coupled receptors (GPCRs): A_1_, A_2A_, A_2B_, and A_3_ ARs. Specifically, A_1_ and A_3_ adenosine receptors (ARs) are coupled to Gi proteins, which inhibit adenylate cyclase (AC), leading to a reduction in cAMP levels. cAMP is located on the intracellular side of the plasma membrane and is important for activating intracellular second-messenger systems. Conversely, A_2A_ and A_2B_ ARs are coupled to Gs proteins, and their activation leads to an increase in cAMP [12].

Under physiological conditions, adenosine is found in low concentrations in the extracellular environment; however, under stress conditions, such as bone loading, fracture, and repair, adenosine concentrations increase [13]. Activation of adenosine receptors has been reported to affect the differentiation and activity of osteoblasts and osteoclasts both in vitro and in vivo, suggesting a role for adenosine in bone healing and regeneration. In particular, activation of A_2A_ ARs has been reported to inhibit osteoclast differentiation through the activation of protein kinase A (PKA) and inhibition of nuclear factor kB (NFkB) nuclear translocation [14], while the selective A_2A_ receptor agonist CGS21680 has been shown to inhibit osteoclast function, by decreasing interleukin-1β (IL-1β) and tumor necrosis factor-α (TNF-α) secretion [15].

Gharibi et al. reported that A_2A_ ARs are expressed in rat MSCs, and their expression is upregulated during the later stages of osteoblastic differentiation, where they play a crucial role in osteoblast maturation and osteoblast phenotype maintenance [1]. Micro-computed tomography of the femur from A_2A_ knockout mice showed a significant decrease in the bone volume/trabecular bone volume ratio, decreased trabecular number, and increased trabecular space. Histological analysis showed an increased number of tartrate-resistant acid phosphatase (TRAP)-positive osteoclasts, and electron microscopy showed increased bone reabsorption [15]. Altogether, these results suggest that A_2A_ ARs regulate osteoclast formation and function in vitro and that deletion of these receptors leads to enhanced osteoclast formation and function in vivo, causing a decline in bone mineral density (BMD) [15].

In vivo, the selective agonist for A_2A_ ARs has been shown to reduce osteoclast-mediated bone resorption in a murine calvaria model of wear particle-induced bone resorption [9]. Micro-computed tomography of calvaria showed that CGS21680, a specific A_2A_ AR agonist, treatment reduced particle-induced bone pitting porosity and increased local bone volume compared to control mice [16]. Moreover, A_2A_ ARs stimulation suppressed inflammation, leading to reduced secretion of pro-inflammatory cytokines and molecules that stimulate osteoclast formation, such as macrophage-colony stimulating factor (M-CSF) and receptor activator of nuclear factor-kB (RANKL) [16].

A number of other studies have been supportive of the role of A_2A_ in bone formation. In a critical size defect model in murine calvaria, Mediero et al. showed that the treatment with the A_2A_ R selective agonist stimulated new bone formation similar to BMP-2 [17]. Zheng et al. reported that local implantation of fibrin gel containing an A_2A_ adenosine receptor agonist enhanced bone healing in rat fractures [18]. Recently, Larranaga-Vera et al. used a conjugate of CGS21680 to alendronate through a PEG-linker to treat osteoporotic mice and showed that mice treated with the A_2A_ AR agonist only exhibited both new bone formation and reduced bone loss [19]. Altogether, these findings suggest a role for A_2A_ ARs in regulating bone homeostasis and regeneration.

In 2002, Varani et al. identified adenosine receptors (ARs) as the primary targets of PEMF stimulation [20]. PEMF exposure significantly increased the density of A_2A_ and A_3_ ARs on the cell membrane of chondrocytes, synoviocytes, and osteoblasts [20]. Notably, A_1_ and A_2B_ receptors are not influenced by the same exposure conditions. Moreover, PEMFs synergize with a specific A_2A_ receptor agonist to elevate intracellular cyclic adenosine monophosphate (cAMP) levels, while an A_2A_ receptor antagonist blocks these effects, suggesting that PEMFs specifically act through A_2A_ adenosine receptors with a pharmacological-like mechanism. In vitro studies conducted in the human osteoblast cell line, hFOB 1.19, showed that PEMF exposure leads to increased expression of A_2A_ and A_3_ ARs, resulting in an increase in cAMP production [21]. The specific A_2A_ agonist, CGS21680, significantly increases hFOB 1.19 cell proliferation, and PEMF treatment further enhances such cellular proliferation. Furthermore, the A_2A_ and A_3_ receptor agonists, CGS21680 and Cl-IB-MECA, respectively, showed anti-inflammatory activity, decreasing the release of inflammatory cytokines and other mediators implicated in bone diseases [21]. This agonist activity inhibits the NF-kB pathway, a key regulator of matrix metalloproteinase (MMP) expression, alongside several inflammatory response genes [6]. However, these effects were blocked when the specific A_2A_ receptor antagonist was present, suggesting that PEMFs act through A_2A_ AR activation via a pharmacologic-like mechanism [20]. An experimental study using an A_2A_ agonist drug demonstrated that PEMF may also provide chondroprotective effects on articular cartilage [22].

Recently Kar et al. studied the role of A_2A_ and A_3_ ARs in PEMF-mediated bone healing by means of gene disruption experiments. The results showed that A_2A_ and A_3_ ARs could activate two complementary signaling pathways involved in PEMF-induced osteoblast differentiation, suggesting that A_2A_ and A_3_ ARs facilitate PEMF action in the initial phases of osteoblast differentiation [4].

Taken together, these results suggest that PEMF-induced bone healing could be mediated at least in part through agonistic activity on A_2A_ ARs. Table 1 summarizes the main findings described in this section. The studies described highlight the significant role played by A_2A_ receptors in bone healing alongside the complementary role played by A_3_ receptors in osteoblast differentiation. These studies show that PEMF activation can activate these receptors.

## 3. Intracellular Signaling and Nuclear Responses to Pulsed Electromagnetic Fields: From Membrane to Nucleus

Intracellular signaling pathways convert received stimuli into cell responses, some of which can be therapeutically advantageous through the reinforcement or repair of local structures [23,24]. This section will describe the intracellular signaling paths, primarily in osteoblasts, resulting in bone formation. Several pathways have been implicated as intracellular messengers of the PEMF signal to the cell-to-bone repair. However, the roles of some of the pathways are still debated and will be briefly discussed to provide a more complete picture.

### 3.1. Calcium/Calmodulin

Several in vitro and in vivo studies showed that PEMF exposure elicits dose–response effects on osteoblast proliferation and on the synthesis of structural and signaling extracellular matrix (ECM) components. An important signaling mechanism described for PEMF involves the release of intracellular Ca^2+^ from the endoplasmic reticulum [25]. The subsequent increase in intracellular Ca^2+^ concentration leads to the activation of the Ca^2+^/Calmodulin pathway, resulting in the upregulation of osteogenic genes, such as Transforming Growth Factor-β family genes (TGF-β1, -β2, -β3), Bone Morphogenetic Protein 2 and 4 (BMP-2 and -4), Fibroblast Growth Factor (FGF)-2, Osteocalcin (BGP), and alkaline phosphatase (ALP) [26]. Activated PLA2 leads to an increase in prostaglandin E2 (PGE2), a potent stimulator of bone formation in vivo and in vitro [27,28].

The increase in intracellular calcium concentration induced by PEMFs has also been reported in hMSCs as an early event during the stimulation of osteogenic differentiation [29]. Currently, the exact mechanism connecting PEMFs, calcium, and osteogenesis remains unclear, likely due to the intricate processes that control calcium influx and the not yet fully understood roles of calcium flux and voltage-gated calcium channels in osteogenic differentiation [30].

### 3.2. Bone Morphogenic Protein

Additionally, PEMF stimulation has been reported to significantly increase the pro-osteogenic activity of members of the TGF-β gene family, including BMP-2 and BMP-4. Martini et al. confirmed the combined osteogenic activity of PEMFs and BMP-2 in human bone marrow MSCs (hBMSCs) [31,32,33] in the presence of low doses of BMP-2. Their findings indicate that the effects of PEMFs were linked to the upregulation of several BMP signaling components, including BMP-2, BMP-6, and BMP type I receptor, and to the activation of SMAD1/5/8, the main player in the canonical BMP signaling pathway [33].

### 3.3. MAPK/ERK and Wnt/β-Catenin

Two major messaging systems that are activated by ARs and result in nuclear activation are the MAPK/ERK pathway and the canonical Wnt/β-catenin pathway.

The mitogen-activated protein kinases (MAPK) pathway transmits extracellular signals, such as those activated by PEMF, to the nucleus by utilizing the three MAPK subunits, composed of serine/threonine kinases: extracellular-regulated kinases (ERKs), Jun N-terminal kinases (JNKs), and p38 [5]. This pathway allows cells to interpret external signals and plays a significant role in many pathophysiological processes, such as differentiation and apoptosis, through the regulation of nuclear transcription factor activation [5]. The MAPK pathway is important for osteogenic differentiation, particularly through its interactions with the TGF-β/BMP gene family.

The canonical Wnt/β-catenin pathway promotes MSC commitment to differentiation into the osteoblastic lineage while indirectly repressing osteoclast differentiation and, accordingly, bone resorption by increasing the secretion of osteoprotegerin [22]. The pathway is stimulated by PEMF and is a regulator of bone homeostasis. A review reported increased bone mass as a result of increased Wnt–β-catenin pathway activation and decreased bone mass as a result of Wnt–β-catenin inhibition [5]. Increases in cAMP, which are further increased by A_2A_ receptors, enhance transmembrane signaling and activate intracellular second-messenger systems such as MAPK and Wnt–β-catenin.

### 3.4. Other Relevant Pathways

Other intracellular signaling pathways of importance have been described as well. Miyamoto et al. described the effects of PEMF on osteoblasts as they related to cellular responses such as the mTOR pathway [2]. They found that intermittent PEMF stimulation may participate in accelerated cell proliferation to promote fracture healing [2]. Wang et al. reported activation by PEMF exposure of the sAC–cAMP–PKA–CREB signaling pathway, stimulating osteogenic differentiation and mineralization [34].

The osteogenic differentiation of bone marrow mesenchymal stromal cells (BMSCs) may be stimulated through PEMF-activation of the Notch signaling pathway [35]. Four Notch receptors have been identified in humans (Notch 1–4), allowing the Notch pathway to play a dimorphic role in bone turnover [36]. Notch signaling on osteoblasts is cell context-dependent and not strictly inhibitory or stimulatory. For example, increased bone formation can result from a restriction of Notch signaling in osteoblasts [36].

Collectively, these data highlight the important role these pathways play in fracture healing, alongside PEMF interactions with second messenger systems and the membrane. Table 2 summarizes the main findings described in this section.

## 4. Pulsed Electromagnetic Field Stimulation of Bone Matrix Synthesis: From Stem Cells to Bone

PEMF has been shown to enhance chondrogenic and osteogenic mesenchymal stem cell (MSC) differentiation, most likely through AR activation [37]. AR activation activates many second messenger pathways, which activate relevant genes within the nucleus, where relevant bone proteins are eventually synthesized.

Some MSC types can express an adipogenic, chondrogenic, or osteogenic phenotype distinguished through the synthesis of molecules such as collagen, proteoglycan, fibronectin, and CD44 [38]. A study found increased osteoblastic gene expression in response to specific PEMFs on human bone marrow-derived MSCs [39]. MSCs are involved in bone repair following an injury, such as a fracture, through endochondral ossification and intramembranous ossification [40]. During endochondral ossification, MSCs differentiate into chondrocytes to create a cartilage model, which is then replaced by vasculature and osteoblasts, which synthesize osteoid and induce calcification into bone. During intramembranous ossification, cartilage is not first formed, and MSCs directly differentiate into osteoblasts instead [40]. In both types of bone formation, osteoblasts build bone by depositing osteoid (the organic bone matrix) during bone remodeling and repair. Bone formation occurs when osteoid is calcified with inorganic calcium hydroxyapatite [41].

Bone morphogenic protein (BMP) and transforming growth factor β (TGF-β) are the primary stimulators of MSC differentiation into osteoblasts and chondrocytes. BMPs have multiple subtypes. A comprehensive analysis found that BMP-2, -6, and -9 were the most potent in the differentiation of MSCs into osteoblasts [42]. Interestingly, low BMP-2 levels are associated with MSC differentiation into adipocytes [43].

**Figure 1:**
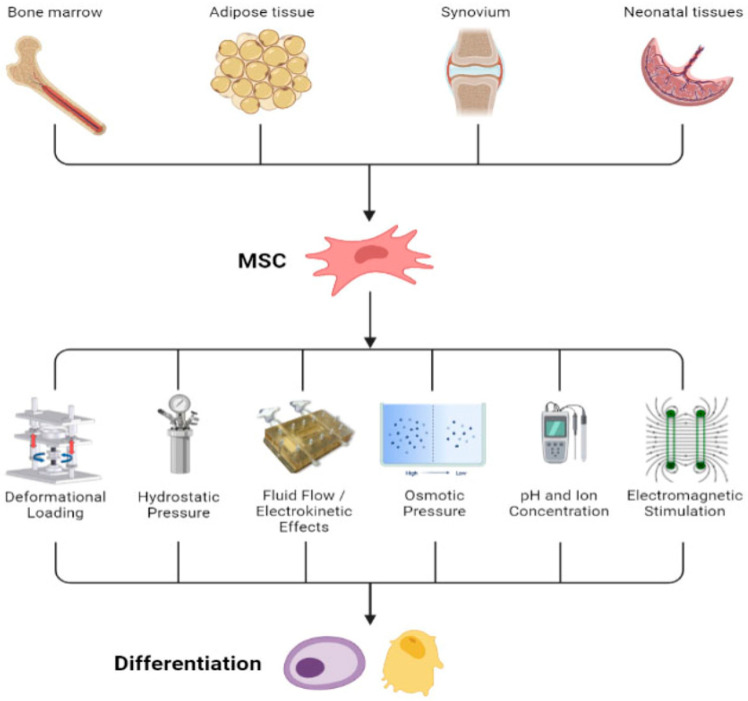
Sources of MSCs and physical factors that can stimulate MSC differentiation into osteogenic cells. Figure from Hung et al. [45].

TGF-β is a potent chemotactic agent that stimulates MSC, pre-osteoblast, osteoblast, and chondrocyte proliferation. During the early stages of fracture healing, TGF-β is released by activated platelets to induce MSC migration and proliferation [44]. Taken together, both BMP and TGF-β are essential for normal fracture healing since they are critical for MSC differentiation into osteogenic cells. Figure 1 below summarizes the impact of several physical factors, including PEMF, on MSC differentiation.

With PEMF exposure, bone marrow-derived and other MSCs promote a more rapid onset of osteogenesis when compared to unexposed controls. This was identified through enhanced alkaline phosphatase, osteocalcin, BMP-2, and TGF-β measurements only during the early stages of differentiation [31,46].

Bone matrix synthesis is stimulated by PEMF through an increase in osteocalcin, alkaline phosphatase, and matrix mineralization in BMSCs and adipose stem cells (AMSCs), according to Ongaro et al. [32]. PEMF has also been shown to significantly enhance alkaline phosphatase production, an early osteogenesis marker, within seven days in “both basal and osteogenic cultures as compared to untreated controls” [39]. In an AMSC and osteoblast co-culture, Ehnert et al. demonstrated that PEMF increased osteogenic differentiation and proliferation [47]. Of particular importance, the Wnt–β-catenin pathway, which is stimulated by PEMF, increases the expression and activation of transcription factors, which, in turn, stimulate the nucleus and protein synthesizing apparatus of subcellular organelles to synthesize the extracellular structural proteins that comprise osteoid.

**Figure 2:**
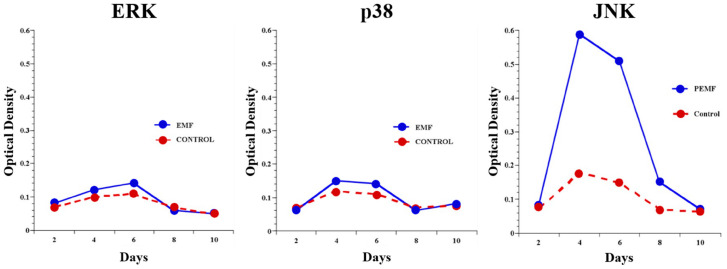
Effects of PEMF exposure on MAPK subunits. This is a western blot analysis of the three MAPK subunits, ERK, p38, and JNK, indicating that JNK is the only subunit affected by PEMF exposure. Figure from Littman [5].

Poh et al. showed that PEMF induced protein kinase B (Akt) and activation of the MAPK/ERK signaling cascade. This significantly upregulated osteocalcin, collagen type I, and alkaline phosphatase levels [48]. ARs activate MAPKs and, thus, provide an intracellular pathway for PEMF signaling. As a result of MAPK activation, PEMF upregulates ECM molecules, collagen type I, alkaline phosphatase, and osteocalcin [48]. Figure 2 describes how JNKs, a MAPK subunit, seem to be the most affected by PEMF exposure.

**Figure 3:**
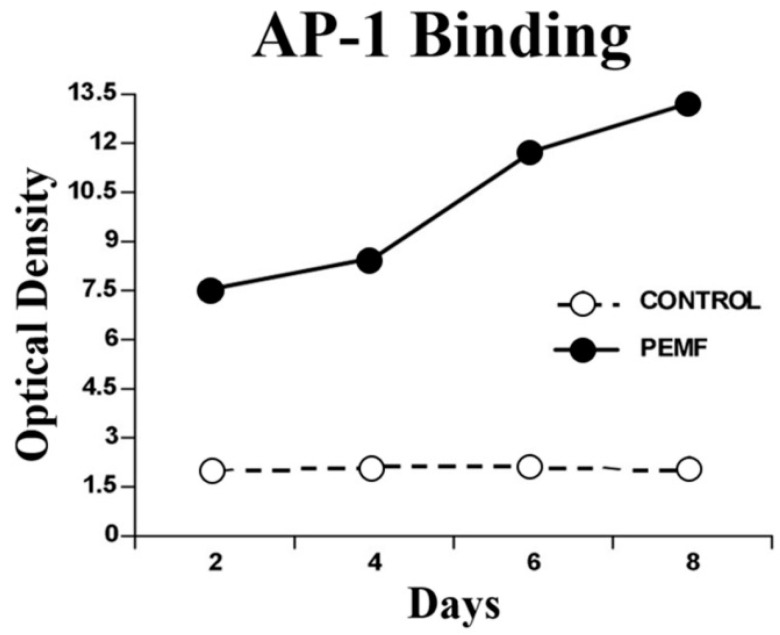
EMSA of nuclear extracts of ossicles demonstrating increased AP-1 binding as a result of PEMF stimulation. Figure from Littman [5].

One of the transcription factors activated by MAPKs is activating protein-1 (AP-1), which controls several cellular responses and regulates gene expression from external stimuli. Notably, AP-1 is a transcription factor for TGF-β, and it is enhanced by PEMF exposure. Electromobility shift assay (EMSA) of nuclear extracts has shown that, in experimental endochondral ossification, PEMF stimulation bound AP-1 at a significantly higher rate at all times compared to non-PEMF-stimulated endochondral bone formation, indicating increased transcriptional activity [5]. These results are described in Figure 3. As a consequence of nuclear activation by AP-1, a variety of structural and signaling proteins that are essential for successful bone healing are synthesized. These proteins include osteoprotegerin, osteocalcin, and collagen type I.

**Figure 4:**
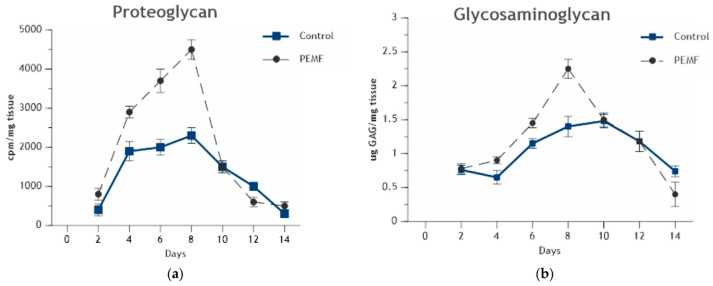
(**a**) In experimental endochondral ossification, PEMF stimulation produced a significant increase in proteoglycan synthesis on day 4 of stimulation, peaking at day 8 before dropping down to normal levels associated with calcification. (**b**) PEMF stimulation gradually increased glycosaminoglycan content between days 4 and 8 prior to calcification onset on day 10. Reprinted with permission from Ref. [45]. 2024, Oxford University Press.

Immediately after a fracture occurs, a hematoma forms, where hematopoietic stem cells, such as MSCs, are recruited to the fracture site. Over a variable time period, depending on the fracture, inflammatory tissue and cartilage are formed, which are replaced by osteoid and, eventually, followed by bony callus formation and, finally, bone remodeling [49]. When fractures do not heal after nine months, without signs of healing for three months, they become known as fracture non-unions [50]. During fracture non-union, endochondral ossification occurs in a clinically insignificant degree, preventing the formation of calcifiable cartilage and eventual bone formation. Exposure to appropriately configured PEMF has been shown to stimulate endochondral ossification. In experimental endochondral ossification, radiolabeled sulfate incorporation into glycosaminoglycan demonstrated increased chondrogenesis. The chondroid matrix content was significantly increased and at an accelerated rate by PEMF (Figure 4a,b). Importantly, for the success of endochondral bone formation, chondrogenesis ceased on time, and the cartilage matrix was removed for calcification and bone formation [45].

Fassina et al. demonstrated in SAOS-1 human osteoblasts that PEMF improved cell proliferation and decorin, fibronectin, osteopontin, types I and III collagen, osteocalcin, and TGF-β [51]. Collectively, the data presented in this section show that PEMF stimulates a variety of structural and signaling molecules of importance to bone formation. Table 3 summarizes the main findings from this section.

## 5. Clinical Evidence for Enhanced Fracture Repair by Pulsed Electromagnetic Field Exposure

Bone fractures and non-union present significant challenges in orthopedic care, often leading to prolonged pain, disability, and increased healthcare costs, requiring interventions beyond traditional methods to promote healing. Traditional methods such as casting, surgical fixation, bone grafting, and pharmacological interventions are effective but can be limited by patient-specific factors, complications, or delayed healing. PEMF is a non-invasive treatment modality that has emerged as a promising adjunct therapy, offering a non-invasive approach to stimulate bone repair [52,53]. Recent reviews suggest that PEMF stimulation is both beneficial and cost-effective for specific orthopedic conditions, particularly when used alongside standard first-line treatments. When applied appropriately, PEMF stimulation enhances the success rates of fracture healing and is effective in preventing and treating non-unions [6,53,54,55]. PEMF stimulation is U.S. F.D.A. approved as a non-invasive method to promote bone healing and is widely used in both the U.S. and Europe.

Evidence for the clinical efficacy of PEMF in bone healing is provided by observational studies, controlled trials, and meta-analyses for both fresh fractures and non-unions.

### 5.1. Clinical Efficacy of PEMF in Fresh Fractures and Osteotomies

Del Buono et al. conducted a case-control study with 50 diaphyseal tibial fractures that underwent reduction and nailing fixation and were allocated to two groups: PEMF-stimulated and unstimulated controls [7]. The pain was significantly lower in the PEMF group at three months, with an average functional recovery of 4.1 months, while control patients took an average of 5.3 months (*p* < 0.0001). The PEMF group achieved fracture healing, assessed by X-ray, more quickly (12.3 ± 2.8 weeks) than the control group (16.5 ± 8.4 weeks, *p* = 0.02). PEMF reduced postoperative pain, analgesic use, and fracture healing time.

In 1986, Fontanesi et al. showed a clinically significant acceleration in healing time in 20 PEMF-stimulated patients (85.7 ± 18.1 days) compared to 20 unstimulated control patients (109.2 ± 30.7 days; *p* < 0.005) [56]. Borsalino et al. conducted a double-blind study of 32 patients (16 treatment and 16 control) who underwent femoral intertrochanteric osteotomy. The authors conducted a roentgenographic evaluation and callus density measurements. All patients were given a placebo control or active PEMF unit on the third day after osteotomy. PEMF exposure accelerated femoral osteotomy healing by increasing callus formation and trabecular bone bridging in the osteotomy area (*p* < 0.01). At 40 and 90 days after surgery, consolidation was described as significantly more advanced in the PEMF-treated group: *p* < 0.05 [57].

Mammi et al. investigated the effects of PEMF in 40 patients treated for degenerative knee arthrosis who underwent valgus tibial osteotomy. Patients were randomly assigned to either a placebo control group or a PEMF-stimulated group. The patients were then rated by osteotomy healing progress into four categories. Category one was the least advanced stage in healing, while category four was the most advanced. 72.2% of the PEMF-stimulated group were ranked categories three and four, while 73.6% of the placebo control group were ranked to categories one and two. PEMF exposure accelerated tibial osteotomy healing (*p* < 0.04) [58].

**Figure 5:**
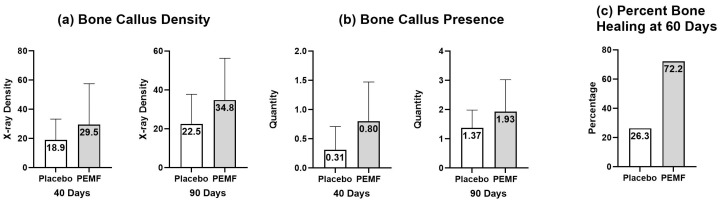
Adapted from Massari et al. [6]. Data are presented as mean +/− standard deviation. (**a**) represents increased bone callus density measurements at both 40 and 90 days with PEMF stimulation compared to placebo from Borsalino et al. (**b**) represents increased bone callus quantity measurements at both 40 and 90 days with PEMF stimulation compared to placebo. (**c**) represents an increased percentage of bone healing at 60 days with PEMF stimulation compared to the placebo from Mammi et al.

In both the Borsalino and Mammi studies, performance bias was minimized by having a standard operating protocol and a single operating surgeon in each [57,58]. The data from both studies were combined by Massari et al. Figure 5 highlights these findings.

Sharrard et al. conducted an RCT assessing the impact of PEMF therapy on tibial shaft fracture, all of which were conservatively treated and had union delay between 16 and 32 weeks [59]. All patients were treated with plaster immobilization; however, 20 patients received active PEMF stimulation units, while 25 received placebo control units for 12 weeks. Radiographic assessments were conducted, and evaluation of the PEMF-stimulated group revealed radiological union in 5/20 fractures, progression toward union in another 5/20, and no progress in 10/20 cases. In the control group, only one fracture showed union, another showed progress, and 23/25 showed no progress. These results indicated a highly significant difference in favor of the active group (*p* = 0.002). The study concluded that PEMFs significantly promote healing in tibial fractures with delayed union.

Faldini et al. conducted an RCT of 77 patients with femoral neck fractures treated with screw fixation in which patients were randomized into a PEMF-stimulated group or a placebo control group. During follow-up at 15.7 months, fracture healing occurred in 15/16 (94%) of patients who were compliant with the active PEMF treatment (more than 6 h/day), compared to 11/16 (69%) in the placebo group. Pain levels were significantly lower in the compliant active group at all follow-up visits compared to the placebo group. In compliant patients, a reduced incidence of osteonecrosis was observed (37% vs. 78%, *p* < 0.03). This demonstrated that PEMF can increase healing rates of fresh femoral neck fractures [60].

In these six studies of the clinical efficacy of PEMF for fresh fractures and osteotomy, PEMF has been demonstrated to increase healing rates by accelerating the healing time and increasing the number of patients who were healed when compared to a placebo control group.

### 5.2. Clinical Efficacy of PEMF in Non-Unions

Marcer et al. completed a case series where a 73% healing rate was observed in 147 patients who underwent PEMF stimulation for 10 h/day after tibial, femoral, or humeral external fixation. On average, the time elapsed since the original fracture was 13.8 months, and an average of 3.3 operations were performed without successful union prior to PEMF-stimulated [61].

Traina et al. reported a controlled study of patients suffering from non-union fresh tibial fractures in which they found that 41 PEMF-stimulated patients had a shorter union time compared to 26 unstimulated control patients. In the control group, the average healing time was 7.8 ± 3.5 months versus 5.7 ± 2.5 months in the stimulated group (*p* < 0.01); 69% of the control patients healed compared to healing in 88% of the PEMF-stimulated patients (*p* < 0.03) [62].

In a prospective comparative study, Cebriàn et al. reported 57 patients who underwent intramedullary nailing for non-union of tibial pseudoarthrosis. Of those patients, 22 received PEMF stimulation in addition to the nailing [63]. Successful healing was observed in both groups. 20/22 (91%) in the PEMF-stimulated group compared to 29/35 (83%) in the surgery-only group. The average time to union, based on radiological evidence, was 3.3 months with PEMF and 4.9 months with the surgery-only group (*p* ≤ 0.05). PEMFs proved beneficial in treating tibial non-union, and their non-invasive nature contributed to a higher rate of complication-free unions.

**Figure 6:**
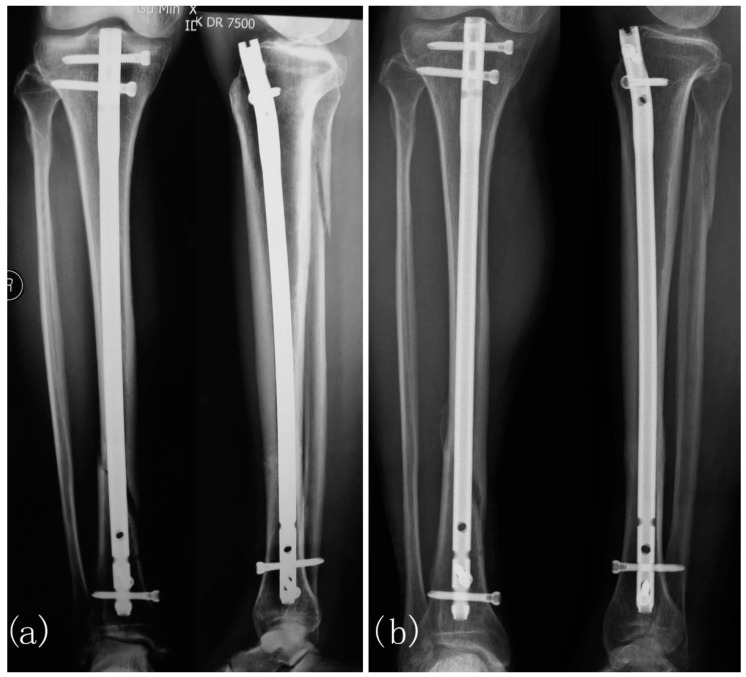
Delayed Union of Tibial Fracture in a 65-year-old Patient Treated with PEMF. (**a**) Delayed union present after closed reduction and intramedullary fixation 16 weeks post-op. PEMF treatment was initiated at this stage. (**b**) Fracture union observed at the 3-month treatment mark. Figure from Shi et al. [64].

Shi et al. conducted an RCT that evaluated the effectiveness of early PEMF application compared to a placebo control group in the treatment of delayed union of long-bone fractures [64]. A total of 58 patients who presented with delayed union ranging from 16 weeks to 6 months were included. Of those patients, 31 received PEMF stimulation, and 27 were in the control group. Clinical and radiological assessments were conducted to evaluate the healing progress. PEMF treatment, administered for an average of 4.8 months, resulted in a 24/31 (77.4%) success rate. This was significantly higher than the control group, which had a success rate of 13/27 (48.1%) with an average treatment duration of 4.4 months (*p* = 0.029) [64]. Figure 6 presents an example of a delayed union of a tibial fracture treated with PEMF.

Murray et al. found that a longer daily PEMF stimulation in 1382 patients with fracture non-unions was associated with a significant reduction in healing time. The group that used PEMF for 9 h per day on average healed within 112 days. This was 76 days faster than the patients who used PEMF for 3 h or less per day, on average, which took them 188 days to heal (*p* < 0.001) [65].

In 2023, Factor et al. conducted an RCT that investigated a novel PEMF device in the treatment of distal radius fractures in comparison to a placebo group. PEMF treatment significantly demonstrated higher union rates (76%) than the placebo group (58%) at 4 weeks as assessed by CT imaging (*p* = 0.02). Additionally, time to cast removal was notably shorter in the PEMF group (33 ± 5.9 days) compared to the placebo group (39.8 ± 7.4 days) (*p* = 0.002) [57]. Additional observations suggested that early application of PEMF therapy during cast immobilization can improve pain, sensation, range of motion, and daily function in patients with distal radius fractures [66].

### 5.3. Clinical Efficacy of PEMF Described in Meta-Analyses

In recent years, there has been a surge in high-quality studies evaluating the effectiveness of PEMFs in fracture healing. These studies have focused on a range of fracture types and patient populations, providing more robust data on the clinical utility of PEMFs. Several meta-analyses have evaluated clinical studies conducted in recent years:

A 2011 meta-analysis by Schmidt-Rohlfing et al. examined the potential effects of electrical stimulation (ES) on bone healing. The meta-analysis included RCTs that focused on the primary endpoint of the ‘rate of bone healing’. A total of 14 RCTs were identified, encompassing 915 patients. Nine of the 14 studies were suitable for inclusion in the meta-analysis, which yielded a cumulative odds ratio of 3.5 with a 95% confidence interval of 1.94–6.3. This systematic review demonstrated a positive effect of ES on bone healing time (*p* < 0.0001) [67].

In 2014, a meta-analysis of RCTs by Hannemann et al. investigated the use of PEMF or low-intensity pulsed ultrasound system (LIPUS) in treating acute fractures in adults. A total of 737 patients from 13 trials were included in the analysis. Analyzing the time to radiological union, the results were heterogeneous, with a significant benefit observed for PEMF in non-operatively treated fractures or fractures of the upper limb (mean difference [MD] = −26.65, 95% CI = −50.38 to −2.91, *p* = 0.03). No studies investigated PEMF stimulation in the lower limb. Additionally, significant evidence was found in accelerating the time to clinical union in acute diaphyseal fractures (MD = −18.27, 95% CI = −34.59 to −1.95, *p* = 0.03). However, no significant differences were found in time to clinical union in acute metaphyseal fractures (MD = 1.31, 95% CI = 11.45 to 14.08, *p* = 0.84) [68].

In 2020, a meta-analysis by Peng et al. aimed to assess the effect of PEMF on fracture healing. Twenty-two studies involving a total of 1468 participants met the inclusion criteria and were analyzed. The pooled results from 14 studies showed a healing rate of 79.7% in the PEMF group, compared to 64.3% in the control group. PEMF was associated with an increased healing rate (RR = 1.22; 95% confidence interval [CI] = 1.10–1.35; I2 = 48) and accelerated healing time (SMD = −1.01; 95% CI = −2.01 to −0.00; I2 = 90%) also based on inverse variance analysis [69].

Bhandari’s group has presented an interesting progression of the evolution of thought on PEMF in three meta-analyses beginning in 2008. The evolving search patterns, outcome variables, and data observations throughout these meta-analyses are important to highlight because they influence the conclusions as to the efficacy of PEMF in bone repair.

The 2008 meta-analysis of RCTs sought to evaluate the effects of ES on long-bone fracture healing [70]. This particular meta-analysis utilized eligibility criteria, which included studies for which PEMF was not indicated, such as pseudoarthrosis and limb-lengthening procedures. The ES devices used in the studies varied considerably. Eight out of the 11 articles that met inclusion criteria utilized PEMF. Adding further heterogeneity, frequencies of PEMF varied widely from 15 to 75 Hz, and the electromagnetic force ranged from 0.0025 to 150 V. The trials used a wide range of daily treatment duration from 4 to 24 h per day over a treatment period ranging from 4 to 76 weeks. The clinical outcomes consisted of a heterogeneous group of markers such as tenderness, pain at specific time points, osteonecrosis rates, arthroplasty needs, radiographic injury severity, pseudarthrosis, and re-displacement rates that compromised statistical assessment. Given the heterogeneity of included conditions and stimulation dosimetry and the wide range of clinical outcomes, there were no benefits for PEMF stimulation use. The authors’ data observations on radiographic outcomes described four studies with a nonsignificant pooled relative risk of 1.76, favoring PEMF. The authors concluded that the impact of PEMF on fracture healing was uncertain and that the current evidence at the time was insufficient in supporting the benefit of PEMF in improving union rates in fresh fracture patients, osteotomy, delayed union, or non-union [70]. It must be noted that given the wide range of eligibility and inclusion criteria applied to the studies, alongside the variety of devices, frequencies, electromagnetic forces, and treatment durations, it is difficult to reach any conclusions.

Six years later, Bhandari’s group conducted a second meta-analysis to indirectly compare low-intensity pulsed ultrasonography (LIPUS) with electrical stimulation (ESTIM) for fracture healing [71]. This time, the authors searched two Cochrane systematic reviews to identify relevant RCTs. A total of 15 eligible ESTIM trials were found. This restricted the inclusion criteria to trials that enrolled patients with more homogeneous and appropriate pathology, either a recent fresh fracture or delayed union or non-union. Of those trials, seven reported union rates as one of the outcomes. The patients were randomly assigned to an ESTIM group or a control group. The trials included were heterogeneous in the dose of ESTIM, especially exposure duration. They used a wide range of daily treatment duration from 4 to 24 h per day over a treatment period ranging from 4 to 26 weeks, with one trial’s treatment period ending when the fracture completely healed. The primary outcome variable was the fracture union rate at 3, 6, and 12 months. The criterion for a successful outcome was four cortices of bridging bone. Low-quality evidence showed a nonsignificant benefit for ESTIM over standard care for non-union populations only at 3 months (RR 2.05, 95% CI = 0.99–4.24) but not for fresh fracture populations (RR 1.23, 95% CI = 0.91–1.66). The study concluded that ESTIM had no significant benefit over standard care in improving fracture union rates. It must be noted that none of the ESTIM trials reported functional outcomes [71].

Two years later, the same group conducted a third meta-analysis, this time investigating 12 studies that reported PEMF use as the therapy method [8]. The rest either used direct current or continuous current stimulation, which may work through different mechanisms. Considerable heterogeneity remained as the trials assessed patients undergoing various conditions, such as spinal fusion, fresh fracture treatment, delayed union/non-unions, or surgical osteotomy, in which the biology may be different. Observational or uncontrolled studies were excluded. Data extracted included interventions, reported outcomes and follow-up times, and loss to follow-up. The primary outcome variables were functional improvement, pain relief, and radiographic non-union. The data observations were different this time, showing a significant improvement in bone healing rates when ES was used in comparison to controls. For pain, the pooled estimate of ES effect showed a statistically significant difference in pain when compared to sham controls (MD on the 100 mm visual analogue scale = −7.67 mm, 95% CI = −13.92 to −1.43; *p* = 0.02; I2 = 0%). For function, the pooled estimate of the ES effect was not statistically significant (MD −0.88, 95% CI = −6.63 to 4.87, *p* = 0.76), though this was only based on two trials. For radiographic non-union, the pooled estimate of ES effect showed a reduction in the relative risk of non-union by 35% and the absolute risk by 15% (RR 0.65, 95% CI = 0.53 to 0.81, *p* < 0.01) when compared to sham controls. The authors concluded that ES may improve radiographic union rates and produce clinically significant, albeit modest, improvements in pain [8].

All three meta-analyses highlighted the need for more trials to establish the efficacy of electrical bone stimulators [8,70,71]. As Bhandari’s group used more appropriate search criteria over time, which included but was not limited to trials that investigated conditions where PEMF is used clinically, the growing evidence supporting the safety and efficacy of PEMF in non-union populations is highlighted. Combined, the discussed clinical studies demonstrate the safety and effectiveness of PEMF in accelerating healing time and increasing healing success of clinical fracture non-unions when the treated conditions and dosimetry were appropriate. Table 4 describes the most important findings from each subsection of this section. These studies show consistent positive outcomes by PEMF in clinical settings, improving bone healing rates and reducing pain.

### 5.4. Potential PEMF Limitations

No therapy is 100% effective. Most studies show that PEMF is approximately 75% effective in the healing of fracture non-unions, as discussed in this section. Even though PEMF has been shown to be highly successful, it has limitations that should be mentioned. First, it cannot be used with people who have implanted electrical devices such as cardiac pacemakers and deep brain stimulators. Its teratogenic effects are unknown; therefore, pregnant women should avoid PEMF therapy until those effects are known.

Optimal dosimetry is not known in terms of amplitude, frequency, or duration of use, and the experimental matrix to determine optimum dosimetry is complex, involving frequency, amplitude, duration, magnetic field strength, and other factors. The dosimetry studies that do exist are generally within clinical parameters. We have found no reports of side effects such as burns or nerve/skin damage from PEMF when clinically appropriate doses have been used.

Another technique for bone repair stimulation is LIPUS, which has shown good clinical results. The 2014 meta-analysis by Bhandari’s group [71], which we discussed, compared LIPUS with ESTIM. The meta-analysis found that there was low-quality evidence suggesting a potential benefit of LIPUS compared to ESTIM in improving union rates at 6 months in fresh fracture populations. However, ESTIM demonstrated better results than standard care in improving union rates at 3 months for cases of delayed union or existing non-union. Another meta-analysis has shown that PEMF stimulation seems to reduce healing time, whereas LIPUS may be useful for fresh fractures [72].

### 5.5. Study Selection Criteria

This was a clinical review that generally followed PRISMA guidelines. As authors, we included two scientists who are experts in adenosine receptor activation by PEMF. Their expertise provided guidance to our study selection. The PI has extensive experience using PEMF for bone restoration and bone matrix restoration. For study selection, we searched PubMed, Undermind.ai, Google Scholar, and references from four meta-analyses included in the analysis of fracture efficacy. Since the intent of this paper was to show the progression of PEMF from the membrane to the organ, we had many search queries, including but not limited to membrane receptor activation, intracellular second messengers, synthesis of extracellular matrix molecules, and clinical bone healing. The inclusion criteria focused on studies that demonstrated strong evidence across a wide range, from basic science experiments to RCTs, and ensured the inclusion of more recent studies. This was conducted to provide a more complete picture of the effects of PEMF. The exclusion criteria focused on disregarding studies without strong evidence and/or had a weak study design. The final outcome of our biblio search has been presented throughout this paper with an overarching and detailed account of how PEMF works and is supported by many studies.

## 6. Conclusions

PEMFs have emerged as a valuable non-invasive therapy for the treatment of fresh fractures, delayed union, and non-union, with a growing body of clinical data supporting their efficacy. Until recently, the mechanisms of PEMF stimulation on bones have not been completely understood. This review described current mechanistic observations, specifically through the effects of PEMF on ARs, notably A_2A_ and A_3_, then described various intracellular signaling pathways that may be stimulated by PEMF. Clinical studies, ranging from early trials to recent multicenter RCTs, consistently demonstrate that PEMFs improve healing rates, reduce time to union, and are particularly effective in cases of non-union where traditional methods have failed.

This review is unique because it presents biological pathways, starting from the cellular scale, followed by the tissue and organismal scale, provides a complete explanation of how PEMF works, and unlocks the “black box” that previously existed. The safety profile of PEMFs is favorable, with no adverse effects reported, and their non-invasive nature makes them an attractive option for patients, surgeons, and healthcare providers alike. Additionally, PEMFs have been shown to be cost-effective, reducing the need for surgical interventions and associated healthcare costs.

As the field continues to evolve, PEMFs are likely to become a standard adjunctive therapy in the management of fractures and non-union. With continued research and innovation, PEMFs have the potential to transform the landscape of orthopedic care, offering hope to patients with challenging bone healing conditions.

## Figures and Tables

**Table 1:** Summary of Studies that Highlight the Role ARs Play in Bone Healing.

Authors	Adenosine Receptor	Results
Gharibi et al. [1]	A_2A_	A_2A_ ARs play a crucial role in osteoblast maturation and osteoblast phenotype maintenance.
Varani et al. [20]	A_2A_ and A_3_	PEMF significantly increased A_2A_ and A_3_ ARs density, but not A_1_ and A_2B_ receptors.
Kar et al. [4]	A_2A_ and A_3_	A_2A_ and A_3_ ARs can activate pathways that enhance osteoblast differentiation through PEMF exposure.
Mediero et al. [17]	A_2A_	Treatment with an A_2A_ receptor selective agonist stimulated new bone formation in a murine calvaria model, similar to BMP-2.
Zheng et al. [18]	A_2A_	Local implantation of fibrin gel containing an A_2A_ receptor agonist enhanced bone healing in rat fractures.
Larrañaga-Vera et al. [19]	A_2A_	CGS21680 conjugate to alendronate promoted new bone formation and reduced bone loss in osteoporotic mice.

**Table 2:** Important Pathways Activated by PEMF Exposure that Increase Bone Formation.

Pathway	Affected Molecules	Outcome
Ca^2+^/Calmodulin	TGF-β1, -β2, and -β3, BMP-2 and -4, FGF-2, BGP, and ALP.	Upregulation of osteogenic genes leading to bone formation.
MAPK/ERK	ERKs, JNKs, p38, and the TGF-β/BMP gene family.	Osteogenic differentiation
Wnt/β-catenin	β-catenin, Wnt, osteoprotegerin	MSC commitment to osteoblastic differentiation and repression of osteoclast differentiation
mTOR	mTOR	Promotion and acceleration of cell proliferation and fracture healing.
Notch	Notch 1–4	Bone turnover

**Table 3:** Summary of Key Studies Showing PEMFs’ Effects on Bone Matrix Synthesis.

Author	Affected Molecules	Outcome
Ongaro et al. [32]	Osteocalcin and alkaline phosphatase	PEMF enhances bone matrix synthesis and osteogenic differentiation in BMSCs and AMSCs.
Ehnert et al. [47]	Alkaline phosphatase	PEMF increased osteogenic differentiation and proliferation in AMSCs and osteoblasts.
Poh et al. [48]	Akt, osteocalcin, collagen type I, and alkaline phosphatase	PEMF activation upregulates ECM molecules and osteogenic markers via the MAPK pathway.
Fassina et al. [51]	Decorin, fibronectin, osteopontin, types I and III collagen, osteocalcin, and TGF-β	PEMF improved cell proliferation in SAOS-1 human osteoblasts.

**Table 4:** The Most Important Clinical Findings in Each Clinical Subsection.

Author	Study Type	Fracture Type	Outcome
Faldini et al. [60]	RCT	Fresh (femoral neck)	PEMF increased healing rates (94% vs. 69%) and reduced pain and osteonecrosis incidence in compliant patients.
Shi et al. [64]	RCT	Delayed union (long-bone)	PEMF treatment had a higher success rate (77.4% vs. 48.1%) compared to the control group (*p* = 0.029).
Aleem et al. [8]	Meta-analysis	Various	ES improved radiographic union rates (RR = 0.65) and reduced pain but had no significant functional improvement.

## Data Availability

Not applicable.
